# Gestational Diabetes Mellitus and Antenatal Corticosteroid Therapy—A Narrative Review of Fetal and Neonatal Outcomes

**DOI:** 10.3390/jcm12010323

**Published:** 2022-12-31

**Authors:** Ivana R. Babović, Jelena Dotlić, Radmila Sparić, Miljana Z Jovandaric, Mladen Andjić, Mirjana Marjanović Cvjetićanin, Slavica Akšam, Jovan Bila, Lidija Tulić, Dušica Kocijančić Belović, Vera Plešinac, Jovana Plesinac

**Affiliations:** 1 Clinic for Gynecology and Obstetrics, University Clinical Centre of Serbia, 11000 Belgrade, Serbia; 2 Faculty of Medicine, University of Belgrade, 11000 Belgrade, Serbia; 3 Department of Neonatology, Clinic for Gynecology and Obstetrics, University Clinical Center of Serbia, 11000 Belgrade, Serbia; 4 University Clinical Centre of Serbia, 11000 Belgrade, Serbia

**Keywords:** gestational diabetes mellitus, antenatal corticosteroids, glycemia, fetus, preterm birth, neonates, respiratory distress syndrome

## Abstract

Background: There, we review the pathogenesis of gestational diabetes mellitus (GDM), its influence on fetal physiology, and neonatal outcomes, as well as the usage of antenatal corticosteroid therapy (ACST) in pregnancies complicated by GDM. Methods: MEDLINE and PubMed search was performed for the years 1990–2022, using a combination of keywords on such topics. According to the aim of the investigation, appropriate articles were identified and included in this narrative review. Results: GDM is a multifactorial disease related to unwanted pregnancy course and outcomes. Although GDM has an influence on the fetal cardiovascular and nervous system, especially in preterm neonates, the usage of ACST in pregnancy must be considered taking into account maternal and fetal characteristics. Conclusions: GDM has no influence on neonatal outcomes after ACST introduction. The ACST usage must be personalized and considered according to its gestational age-specific effects on the developing fetus.

## 1. Introduction

Gestational diabetes mellitus (GDM) is any degree of glucose intolerance with the onset or first recognition during pregnancy. Diet modification presents the first-line therapy for the treatment of GDM [1]. It is one of the most common metabolic disorders during pregnancy [2]. The association of maternal hyperglycemia during pregnancy and fetal outcomes of preterm as well as term newborns is still not clear enough. 

There are controversial reports about GDM as a risk factor for prematurity [3]. Therefore, it is impossible to confirm an accurate link between the risk of preterm birth and maternal poor glycemic control. Further, there are controversial results about the increased frequency of respiratory distress syndrome (RDS), respiratory infections, and intraventricular hemorrhage (IVH) in preterm neonates, associated with gestational age at birth in pregnancies complicated by maternal GDM [4]. FIGO (2019) recommends antenatal corticosteroid therapy (ACST) for women who have pre-gestational and gestational diabetes and that are at risk of imminent preterm birth. Moreover, it is recommended that women with prescribed ACST should also receive additional insulin and have close monitoring until the end of pregnancy [5].

In this narrative review, we aimed to show current findings about the pathogenesis of GDM, the pathological influence of GDM on the fetal organic system, and the need for ACST in preterm and term births complicated by GDM, as well as its influence on short-term and long-term neonatal outcomes.

## 2. Materials and Methods

The authors searched the available data on GDM, the influence of GDM on the fetal organic system and neonatal outcomes, as well as the usage of ACST in pregnancies complicated by GDM. The authors searched PubMed, Scopus, The Cochrane Library, and Web of Science for articles available in full and written in English in the period of 1990–2022 in peer-reviewed journals. For literature search, we used a combination of keywords: “gestational diabetes mellitus”, “antenatal corticosteroids,” “glycemia”, “fetus”, “preterm birth,”, “neonates”, “neonatal”, “respiratory distress syndrome”, “intracranial hemorrhage”, “outcome”, and “treatment”. Studies regarding insulin-dependent DM without or with complications during pregnancy, manuscripts not available as full text, or not written in English were excluded from the study. Manuscripts considered by the authors as the most important to the topic were selected for this narrative review.

The findings of the literature search are presented in the Section 3 along with the discussion to illustrate current findings about the pathogenesis of GDM, the influence of GDM on fetal organ systems, and neonatal outcomes, with an emphasis on the usage of ACST in pregnancies complicated by GDM.

## 3. Results and Discussion

### 3.1. Epidemiology and Etiology of GDM

The occurrence of GDM varies in different regions worldwide, from 1% to 45% [6,7]. In the Republic of Serbia, the prevalence ranges from 1% to 14%, and the development of GDM has been reported to be related to advanced age, gynecological alterations, nutritional status, diagnostic criteria, the presence of diabetes in the family, and diabetogenic factors (e.g., obesity, polycystic ovary syndrome) [6,7,8]. GDM is defined as a type of diabetes that is caused by maternal, placental, and fetal factors. It may originate from specific gene mutations, dysregulation of placental hormones, and/or β-cell injury.

GDM usually develops after the second trimester of pregnancy, between the 24th and 28th weeks of gestation. A specific metabolic modification occurs during pregnancy, before the formation of a functional placenta. Insulin resistance (IR) increases during a diabetic pregnancy, and the maternal pancreatic β-cell mass expands due to both hyperplasia and hypertrophy within islets, which enhances insulin secretion. An increase in maternal insulin sensitivity is caused by maternal hyperglycemia, lipogenesis, and lipid storage in adipose tissue [9]. However, in the second and third trimesters, maternal insulin sensitivity decreases and induces an acceleration of fetal growth in normal pregnancy. Significant maternal lipolysis and gluconeogenesis are also observed [10].

### 3.2. Pathogenesis of GDM

There is evident placental control of maternal metabolism and cardiovascular homeostasis. Placental progesterone and estrogens are key steroid hormones that control insulin sensitivity during pregnancy [11]. Both steroids induce hypertrophy in the pancreas. Progesterone reduces insulin-stimulated glucose uptake and stimulates appetite and fat deposition, whereas estrogen stimulates systemic insulin sensitivity [11]. Other placental factors, such as human chorionic gonadotropin (HCG), human placental lactogen (hPL), along with human placental growth hormone (PGH), can also modulate metabolic functions during pregnancy and cause GDM [12]. For example, hPL and PGH upturn gluconeogenesis and lipolysis in the liver, while maternal insulin-like growth factor I (IGF-1) levels rise due to increased PGH levels [12]. Furthermore, fine maternal metabolic control depends on the release of prolactin (PRL), which induces β-cell proliferation and insulin secretion [12].

Furthermore, adipose tissue produces adipocytokines, such as leptin, adiponectin, tumor necrosis factor alpha (TNF-α), interleukin-6 (IL-6), and apelin [13]. These hormones play roles in glucose homeostasis and, thus, contribute to IR during pregnancy [13]. Adipose tissue and the placenta produce a similar cytokine pattern, which explains why obese women have a higher risk of developing GDM [13].

Between the 20th and 24th weeks of gestation, the levels of estrogen, progesterone, cortisol, and hPL are elevated in the maternal circulation and this is associated with an increase in IR [14]. Changes in the production of placental hormones, especially progesterone and estrogen, have been correlated with GDM development [14].

GDM onset may also be influenced by mutations in the genes that code insulin, insulin receptor, insulin-like growth factor-2, glucokinase, prolactin growth factor (PRL-GF), and plasminogen activator inhibitor 1 (PAI-1) [14].

However, it is assumed that β-cell injury is the main causative factor for GDM. There is an observed decrease of 41% in the number of β cells in GDM mothers [15]. It has also been found that more than 50% of pregnant women older than 30 years present with GDM [16]. The effect of gravidity on the risk of developing GDM has also been studied; the risk increases with the number of pregnancies, especially in women of advanced maternal age [16]. Obesity is frequently present in women with GDM and it worsens maternal/fetal metabolic alterations [16].

Finally, physiological pregnancy is characterized by low-grade inflammation. This is exacerbated in GDM cases and IR might develop from this pro-inflammatory state [5]. In fact, a pro-inflammatory pattern of upregulated adipokines (i.e., IL-6 and hs-CRP) and a reduction in the anti-inflammatory adiponectin was observed in adipose tissue from women with GDM [17].

### 3.3. Maternal Metabolism in GDM

During the second and third trimesters, a decrease in insulin sensitivity results from an increase in maternal adipose tissue and the release of placental hormones, such as estrogens, progesterone, and hPL [18]. Dysregulation of maternal lipid metabolism begins in early pregnancy before dysregulation of carbohydrate metabolism [19]. The hPL lipolytic effect contributes to the increase in circulating fatty acids [19]. Finally, maternal metabolism shifts toward greater use of lipids rather than glucose as an energy source [19]. High levels of fatty acids in the maternal circulation lead to the development of IR [20]. GDM occurs when the increase in pancreatic β-cell secretion is not able to compensate for this IR [20].

During a normal pregnancy, decreased albumin and increased globulin levels are observed in the maternal serum [21]. Further, hPL and HCG suppress the deamination of amino acids in the liver [21], and amino acids are preserved as the fetal source of energy and for fetal development [21]. The maternal–fetal amino acid interface is mediated by active transport processes (i.e., enzymatic mechanisms mediated by ATPase), and at least 15 different amino acid transporters have been identified [22]. System A transporter is the most involved molecule; it facilitates the uptake of small, non-essential, neutral amino acids (i.e., alanine, glycine, serine) from the maternal circulation and their delivery to the fetal circulation [22]. The concentrations of amino acids are generally higher in the fetal circulation than in the maternal circulation, reflecting this active transport [22].

The first trimester of pregnancy is characterized by an increase in maternal energy reserves, and this is mainly mediated by lipogenesis and results in the storage of substrates that will be required during advanced gestation and breastfeeding [22]. In contrast, during the second and third trimesters, there is a significant reduction in insulin sensitivity and an increase in maternal concentrations of glucose and free fatty acids [22]. This period of pregnancy is characterized by a progressive decrease in fasting glucose and an increase in maternal hepatic gluconeogenesis [22,23].

In cases of GDM, there is evidence of relationships between GDM and impaired maternal glucose metabolism and plasma lipid metabolism, especially in older and obese women. While some newer studies have found that GDM induces a state of dyslipidemia that is consistent with IR, the relationship remains unclear. Hu et al. [24] found significantly higher triglyceride (TG) levels in women with GDM compared to women without GDM, and the levels were found to be associated with maternal race, ethnicity, and obesity [24,25]. Wang et al. [26] reported that the TG/HDL ratio recorded during the first trimester is linked to the risk of developing GDM. The authors concluded that women with GDM have significantly higher levels of circulating TG, total cholesterol (TC), low-density lipoprotein-C (LDL-C), very-low-density lipoprotein-C (VLDL-C), and lower levels of high-density lipoprotein (HDL-C) compared to women without GDM [26]. Anderson et al. [27] found that higher levels of phosphatidylcholines and lysophosphatidylcholines are correlated with GDM [27].

Clearly, GDM is a multi-faceted condition that is affected by and impacts the function of many metabolic pathways, including carbohydrate, lipid, and amino acid metabolic pathways. Given the complexity of the condition, the specific roles that these pathways play in the development and persistence of GDM remain obscure. Research has, however, revealed that dysfunction of the plasma amino acid metabolism is associated with increased oxidative stress and the perturbation of antioxidant mechanisms [28]. Further, it has been found that alanine can transitorily reduce glucose levels by altering cellular energy metabolism in diabetic mothers with poor glycemic control [29]. Furthermore, increased plasma arginine concentrations in women with GDM have been found to be related to decreased uptake of adenosine by endothelial cells [29]. Thus, this could be an etiological factor of the endothelial dysfunction observed in GDM patients [30].

The placenta is also particularly sensitive to maternal hyperglycemia throughout pregnancy, and it directly contributes to fetal macrosomia [31,32]. The risk of fetal macrosomia is closely related to the maternal control of glycemia in GDM. Macrosomia is diagnosed when the full-term birth weight is >4000 g or when, at any gestational age, the birth weight is above the 90th percentile of the corresponding birth weight [33]. The authors suggest that sonographic control of fetal growth should start between the 24th and 28th weeks of gestation [34]. Fetal macrosomia has been found to be related to fetal leptin. Leptin is an amino acid product secreted by adipocytes; however, throughout pregnancy, leptin is mostly produced by the trophoblast. The exact roles of leptin and C-peptide in the control of fetal growth are not yet clear [35].

### 3.4. Fetal Metabolism in GDM

The fetus requires proteins as structural components and glucose as an energy source [36]. Amino acid transport through the placenta also presents an important parameter of fetal growth. In research, GDM is usually connected to increased system A and L amino acid transport activity, and this activity is modified by pro-inflammatory cytokines, such as TNF-α and IL-6 [37]. Thus, GDM may enhance amino acid transport across the placenta. Kadakia et al. [38] found that the presence of aromatic amino acids (AAAs), such as tyrosine, and their metabolic products in cord blood are positively associated with neonatal birth weight [38].

A disturbance in maternal lipid metabolism is related to a disturbance in fetal lipid metabolism in pregnancies complicated by GDM. Fatty acids are essential for fetal development because they are used as structural components and a source of energy [39]. In fact, all fatty acids can provide energy [39]. Polyunsaturated fatty acids (PUFAs) of the n-3 or n-6 series are mainly required for structural and metabolic functions. Mitochondrial fatty acid oxidation occurs in fetal tissue and the placenta and contributes to energy production. From the 25th week of gestation in humans, the fetal buildup of lipids and fatty acids rises logarithmically according to gestational age [39]. Fetal hyperinsulinemia in GDM patients supports surplus deposition of adipose tissue, which induces altered adipocytokine production. Fetal lipoprotein levels are low at birth, but the effect of GDM on these levels is unclear. The augmentation of body fat observed in the neonates of women with GDM is a risk factor for obesity in early childhood and later life. In premature infants, arachidonic acid (AA) has specific importance for growth and development. The growth-promoting effect of AA might be connected to its function as a precursor of prostaglandins as well as its structural role in membrane phospholipids. The fetus presents with decreased lecithin–cholesterol acyl-transferase activity, which results in low plasma cholesterol esterification [40].

### 3.5. GDM and Premature Birth

An association between GDM and maternal obesity has been confirmed, and an increased frequency of preterm labor is found in obese women with GDM. Maternal obesity related to hyperglycemia (especially in the second trimester), lipotoxicity, IR, and oxidative stress leading to endothelial dysfunction in the placenta and preeclampsia is one of the major etiological factors in preterm labor. Berger et al. [41] determined that the highest risk for preeclampsia development is observed in women with GDM complicated by hypertension and obesity mutually. GDM is related to an increased occurence of poor perinatal outcomes, chronic placental insufficiency, chronic hypoxia, premature birth, and fetal macrosomia [42,43]. It is also linked to perinatal complications, which are more frequent among premature newborns, such as respiratory distress syndrome, hypocalcemia, and hypoglycemia [44]. The relationship between maternal hyperglycemia, inflammatory changes in placental vessels, and oxidative stress is well known in GDM. It is not clear, however, whether the key factors that contribute to preterm delivery and prematurity of infants are connected to GDM. The association of maternal hyperglycemia during pregnancy and the outcomes of premature infants are also unknown [43].

As an anabolic hormone, insulin is one of the factors regulating fetal growth. Maternal hyperglycemia during pregnancy can negatively impact fetal insulin production from the 24th week of gestation. It has been found that during the second trimester, the fetuses of women with GDM have prominent β-cell masses in the pancreatic islets and release additional insulin after acute exposure to glucose compared to the fetuses of nondiabetic women [44]. These features become even more noticeable in later gestational age. In addition, the C-peptide level in umbilical blood, which is a marker of fetal hyperinsulinemia, was found to correlate with infant weight in women with GDM. Indeed, fetal macrosomia can be caused by maternal-to-fetal glucose transfer through aberrant expression and functioning of glucose transporter proteins (Glut proteins) in the placenta in pregnancies complicated by GDM [44]. Fetal macrosomia in diabetic mothers can be managed by adequate glycemic control throughout pregnancy. Chiefari et al. [45] showed that steps taken in order to reduce fetal macrosomia in GDM patients should be initiated between 16 and 18 weeks [45].

Insulin resistence is mediated by pregnancy-associated hormones (e.g., estrogen, progesterone, cortisol, cytokines, and growth hormones) that are produced in the placenta and enter the maternal circulation, such as PGH and hPL. Adipose tissue creates adipocytokines, such as leptin, adiponectin, and TNF-α, that potentially contribute to IR. Generally, IR causes glucose intolerance, which brings about hyperglycemia. This leads to alterations in placental function and excessive levels of glucose, amino acids, and lipids in the fetus. In addition, PGH expedites fetal gluconeogenesis and lipogenesis, contributing even more to augmented fetal growth.

Contrary to maternal hypoglycemia, fetal growth restriction (FGR) occurs in GDM pregnancies. Tarry-Adkins et al. [46] reported that term infants whose mothers with GDM were treated with metformin had children whose birthweights were, on average, 108 g smaller than those whose GDM mothers given insulin [46]. No major differences were noticed in head circumference between the children of diabetic mothers and those of the healthy controls. Infants from mothers with GDM had more intensive-care unit admissions (OR 4.89 and 1.68, respectively) because of respiratory morbidity. Battarbee et al. did not find any significant correlation of maternal GDM and neonatal necrotizing enterocolitis and intraventricular hemorrhage and grade 3 or 4 [47]. Hitaka et al. investigated very-low-birthweight (VLBW; <1500 g) infants in Japan and showed that maternal hyperglycemia in pregnancy was not a key factor in neonatal mortality and morbidity. In the same study, a higher risk for RDS was registered only for infants of mothers with hyperglycemia during pregnancy [48]. Pregnancy complicated with diabetes is linked to a greater risk of neonatal respiratory morbidity at all weeks of gestation. Fetal hyperglycemia and hyperinsulinemia have been proposed to present the core of the mechanisms causing delayed pulmonary maturation. Delayed finding of phosphatidylglycerol in the amniotic fluid, which is a marker of lung maturity, was found to be connected with inadequate glycemic control [49]. Kawakita et al. found that insulin leads to a decrease in surfactant protein gene transcription in human lung epithelial cells [50]. Pregnant women with preexisting diabetes mellitus were found to exhibit a postponement in fetal surfactant system maturation, due to a substantial rise in fetal plasma glucose and insulin levels compared to women with GDM. This may explain the higher incidence of neonatal RDS in women with preexisting diabetes compared to women with GDM. Since diabetes-related neonatal respiratory morbidity was not completely explained by prematurity-associated physiological immaturity, the authors concluded that the actual risk factor might be diabetes itself [48,49,50].

### 3.6. GDM and the Infant’s Cardiovascular and Nervous Systems

Myocardial hypertrophy is observed in fetuses and neonates of patients with preexisting diabetes mellitus and GDM [51]. In both groups, the etiological factor is unclear. It can introduce hyperinsulinemia as well as hypoxia. One of the first signs of fetal myocardial hypertrophy is increased intraventricular septa, and a later sign is decreased diastolic function. Although clinically asymptomatic, myocardial hypertrophy is most frequently diagnosed during the third trimester in patients with GDM [51]. Babovic et al. found an association of inadequate maternal glycemic control (HbA1c ≥ 6.1%) and interventricular septal thickness [52]. Neonatal macrosomia and poor maternal glycemic control do not correlate with neonatal myocardial hypertrophy. Hence, it could be concluded that myocardial hypertrophy is transient in mothers with GDM [53].

The weight of the placenta is increased in GDM patients, and this may be due to maternal and fetal hyperglycemia, fetal hypoxia, and poor neonatal adaptation [50]. Antoniou et al. [54] investigated the offspring of 576 pregnancies of women with GDM and found that only 10.7% exhibited neonatal hypoglycemia. The incidence of neonatal macrosomia in women with GDM depends on which diagnostic criteria are applied for GDM screening (e.g., the Diabetes and Pregnancy Study Group (IADPSG) versus the National Institute for Health and Care Excellence (NICE) in the United Kingdom). A study showed that mild unregulated maternal hyperglycemia estimated by both criteria was linked with increased birth weights in GDM pregnancies [55].

Development of the fetal brain occurs during the course of the whole pregnancy. The functioning of cerebral hemispheres can be negatively impacted by GDM-induced metabolic disturbances during the second half of pregnancy. Inadequately treated GDM can lead to substantial metabolic dysfunction, which, in turn, can result in a certain degree of developmental disorders in the children [56].

### 3.7. Antenatal Corticosteroids (ACSTs) in GDM Pregnancies

Weight gain is not unusual during pregnancy and it is manifested by the deposition and hypertrophy of maternal adipocytes. Being overweight and obese are the major risk factors for GDM. Lau et al. [57] confirmed that pre-pregnancy body mass index (BMI) is one of the central risk factors for the development of GDM [57]. Moreover, being overweight or obese during pregnancy, as well as having GDM, can increase the posibility of having adverse outcomes, such as metabolic disorders, premature delivery, hypertension, and stillbirth [58].

RDS is the principal reason for morbidity and mortality in preterm-born children. Glucocorticoids cross the placenta and boost fetal pulmonary maturation and surfactant production [59]. ACSTs have been shown to reduce RDS when administered after the 26th week and before the 35th week of gestation and to reduce IVH and neonatal death when administered after the 26th week and before the 30th week of gestation. Ehret et al. [60] showed that ACSTs applied between the 23rd and 24th gestation weeks did not reduce neonatal IVH, leukomalacia, neonatal enterocolitis (NEC), and later neurodevelopmental delays 18–24 months after birth [60]. Late preterm infants (born after the 34th to 36th gestational week) generally have a very low risk of prematurity. Gyamfi-Bannerman et al. [61] reported a greater risk of respiratory and neurodevelopmental complications in these infants in comparison with term infants. Betamethasone administered as two 12 mg intramuscular injections 24 h apart between the 34th and 35th gestational weeks reduced neonatal transient tachypnea but not RDS [61]. Kerstjens et al. [62] noted increased neonatal hypoglycemia (2.2 mmol/L) as an independent factor in neurodevelopmental delay in these infants [62]. This is one of the reasons for inconsistent recommendations for ACSTs in such pregnancies. Administration of ACSTs 24 h before delivery in early-term elective cesarean sections (37–38 gestational weeks) is not correlated with a noteworthy decrease in RDS risk. The risk in this gestational week is generally low (about 2.7%) compared to the risk of increasing the exposure of the population to ACSTs from about 10% to 20% [63]. The FIGO guideline supports optimal timing for the use of ACSTs in preterm labor, which is between 24 and 34 weeks gestation, when the birth is anticipated within the next 48 h, with benefits detected for up to 7 days [5].

### 3.8. ACSTs, Glucose Metabolism in Preterm Infants, and Programming of Maturation

Prematurity increases the risk of hypoglycemia. Therefore, in current protocols, administering ACSTs close to delivery is suggested, when maternal hyperglycemia presents the highest risk of neonatal hypoglycemia. The risk of neonatal hypoglycemia is espetially relevant in women with GDM who are at risk of preterm delivery and more frequent fetal immaturity at any given gestation [64]. It is well known that neonatal hypoglycemia associated with GDM is the consequence of neonatal hyperinsulinemia, as well as maternal hyperglycemia or possibly the suppression of the neonatal hypothalamic–pituitary–adrenal stress axis. Rowe et al. [65] recommended intravenous insulin administration after betamethasone therapy to prevent maternal hyperglycemia and neonatal hypoglycemia [65].

Another approach recommends that the dose of ACSTs should be weight-adjusted, because it can lead to more severe and prolonged maternal hyperglycemia in women with a lower BMI [66]. Another study suggested that the rate of maternal hyperglycemia after ACST administration can be reduced by altering the timing of ACST administration and the dosing interval [67]. In GDM, 12 mg doses of betamethasone twice a day could be preferred over 6 mg doses of dexamethasone four times per day. Some literature data indicate that that hyperglycemic episodes were scarcer on the second day after birth, regardless of the term when the former dosage was used [68]. Finally, neonatal hypoglycemia in the late-preterm and term period is a serious condition, but it is not correlated with adverse neurodevelopmental outcomes. The critical cut-off glucose concentration below which neurological injury occurs is still unknown. Substantial hypoglycemia has been linked to adverse outcomes in moderately preterm infants (32–36 weeks of gestation) [69].

### 3.9. ACSTs in the Development of the Fetal and Preterm Neonatal Brain

Endogenous glucocorticoids are known to decrease growth and stimulate cellular differentiation. They are mostly at low levels until just before birth. Krispin et al. [70] showed that neonatal lower birthweight was related to the administration of ACSTs before 34 weeks of gestation [70]. In contrast, fetal hyperglycemia and hyperinsulinemia promote neonatal macrosomia. Studies conducted in animals have shown that ACSTs alter myelination of the fetal nervous system, the hypothalamic–pituitary–adrenal axis, glucose metabolism, and blood pressure [71]. Key risk factors for neonatal hypoglycemia include preterm birth, neonatal macrosomia, and, rarely, neonatal hypotrophia and GDM. The risk of developing hypoglycemia peaks in the first hours postpartum [72]. Shah et al. showed that neonatal hypoglycemia correlates with specific cognitive dfects during early childhood (2–5 years), including a two- to three-fold higher risk of visual–motor deficiency and executive dysfunction [73]. The occipital cortical area responsible for visual and motor functions is particularly susceptible to hypoglycemia because of its higher metabolic activity [74].

ACSTs have been shown to have anti-proliferative effects on neural progenitor cells in the fetal brain. The 34th week of gestation is crucial for brain development, as at that time, brain weight is only 65% of the term brain weight while gyral and sulcal formation is still incomplete [75]. The hypothesis that ACSTs affect neurogenesis and gliogenesis [76] was proven by a 2014 review, which showed that even a single course of ACSTs can lead to a 9% decrease in head circumference (in comparison with both preterm and term unexposed infants) [77]. The incidence of attention deficit hyperactivity disorder (ADHD) is higher among children of mothers who had GDM, especially with complications (hypertension) [78].

### 3.10. ACSTs and the Fetal and Preterm Neonatal Cardiovascular System

Cardiomyocyte hyperplasia occuring shortly before and after birth is found to be linked to structural, functional, and biochemical maturation. These notable cardiomyocyte alterations are caused by mechanical and hormonal elements and are esential for postnatal survival. Fetal systolic and diastolic function improves prior to and just after birth, with advancing maturation of the contractile and relaxation properties of cardiomyocytes.

In preterm infants, heart mass (relative to body weight) is decreased at birth, as well as cardiac load, oxygenation, and arterial pressure. In addition, myocyte hypertrophy can compensate for having less cardiomyocytes to maintain heart mass. In adults who were born preterm, fibrosis may be diagnosed secondary to cardiomyocyte loss [79]. Glucocorticoids are esential for normal maturational changes happening in cardiomyocytes before birth [80]. ACSTs reduce cardiomyocyte proliferation and promote hypertrophy in both the fetal and neonatal heart. In contrast, Aye et al. [79] suggested that preterm birth impacts cardiac morphology and function independently of antenatal corticosteroid therapy. In cases of GDM with poor glycemic control (HbA1c ≥ 6.1%), decreased diastolic function is noted. A single course of antenatal betamethasone therapy was not found to have efects on the cardiac wall thickness and systolic function in premature infants exposed to ACST between 30 and 32 weeks of gestation [81]. Increased blood pressure, reduced human cardiac afterload, and increased fetal cardiac output have been noted; however, insignificant changes in diastolic function follow courses of ACSTs in fetuses. In contrast, concerning the impact of ACSTs on heart development, studies indicate that transient cardiac hypertrophy in preterm neonates occurs if glucocorticoids are administered after birth [81].

According to the FIGO recommendations (2019), administration of ACSTs is an option for women with GDM that are at risk of imminent preterm labor. Women who receive antenatal steroid therapy should also be given additional insulin according to the current protocol, along with close monitoring [5].

## 4. Conclusions

GDM does not modify the neonatal effects of ACSTs, which have gestational age-specific consequences on the developing fetus. This corresponds with the idea that neonatal organs, including the brain and heart, have developmental windows of sensibility to the maturation caused by glucocorticoids that are reliant on the level of organ development: organogenesis, growth, or maturation. The literature indicates that to optimize maternal and fetal outcomes and to avoid severe hypoglycemia in GDM pregnancies, doses of ACSTs should be weight-adjusted. Moreover, the level of maternal hyperglycemia after the administration of ACSTs may be reduced by altering the timing and dosing intervals. Betamethasone may be more effective in GDM pregnancies than dexamethasone. Consequently, in the future, the administration of ACSTs must be individualized and population-based approaches should be discontinued. Nevertheless, there is a need for more studies that focus on ACSTs to confirm current findings about the use and effects of ACSTs in GDM.

## Data Availability

Not applicable here.
